# Supplementary material for: Structural definition of a pan-sarbecovirus neutralizing epitope on the spike S2 subunit
Source: Commun Biol. 2022 Apr 11;5:342. doi: 10.1038/s42003-022-03262-7 (PMC9001700; doi:10.1038/s42003-022-03262-7)
Supplement: Supplementary file 2 — Supplementary Material [file 42003_2022_3262_MOESM2_ESM.pdf]

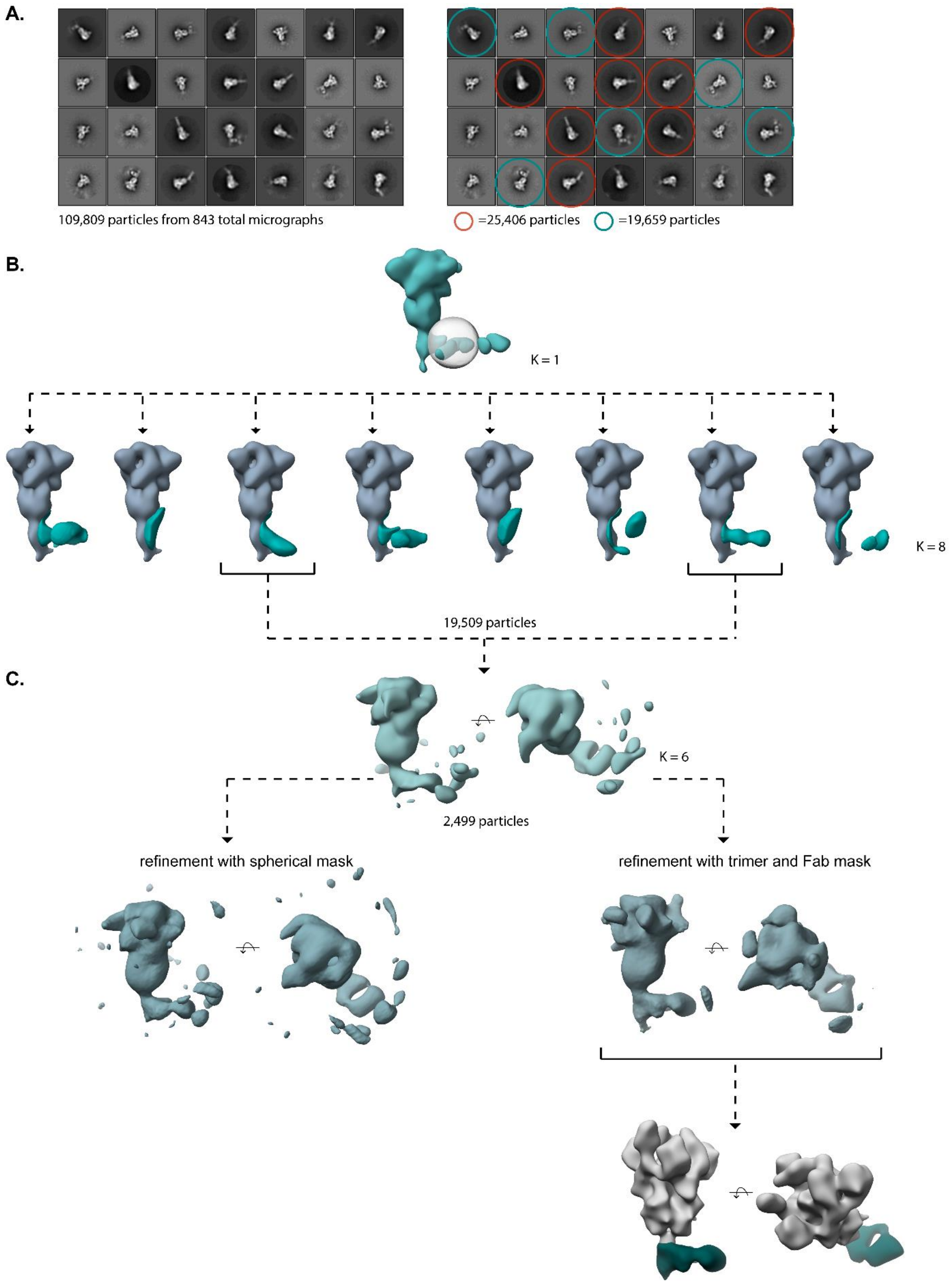

A.
109,809 particles from 843 total micrographs
=25,406 particles
=19,659 particles
B.
K = 1
K = 8
19,509 particles
C.
K = 6
2,499 particles
refinement with spherical mask
refinement with trimer and Fab mask

Supplementary Figure 1. Data processing workflow for CV3-25 IgG in complex with SARS-CoV-2 Spike. (A) Negative stain electron microscopy 2D class averages of CV3-25 IgG bound to SARS-CoV-2 6P-D614G S protein. Unbound trimers with evidence of a long stem at the base of the trimer—circled in red—were used to generate an 3D model. A subset of clean 2D classes with evidence of antibody-bound trimers—circled in teal—were refined using the apo trimer initial model and subjected to C3 symmetry expansion. **(B)** A 40 Å spherical mask was centered on the expected Fab region on the lower S2 domain of one protomer, and 3D classification without alignment on the symmetry expanded particle stack was performed to isolate those particles with clear Fab density. K: number of classes requested during classification run. **(C)** Particles from two focused classification classes were selected, duplicate particles removed, and refined with no symmetry. A final refinement was performed with either a 320 Å spherical mask or a mask of the trimer and Fab to eliminate noise from unbound portions of IgG and better align the Fab signal.

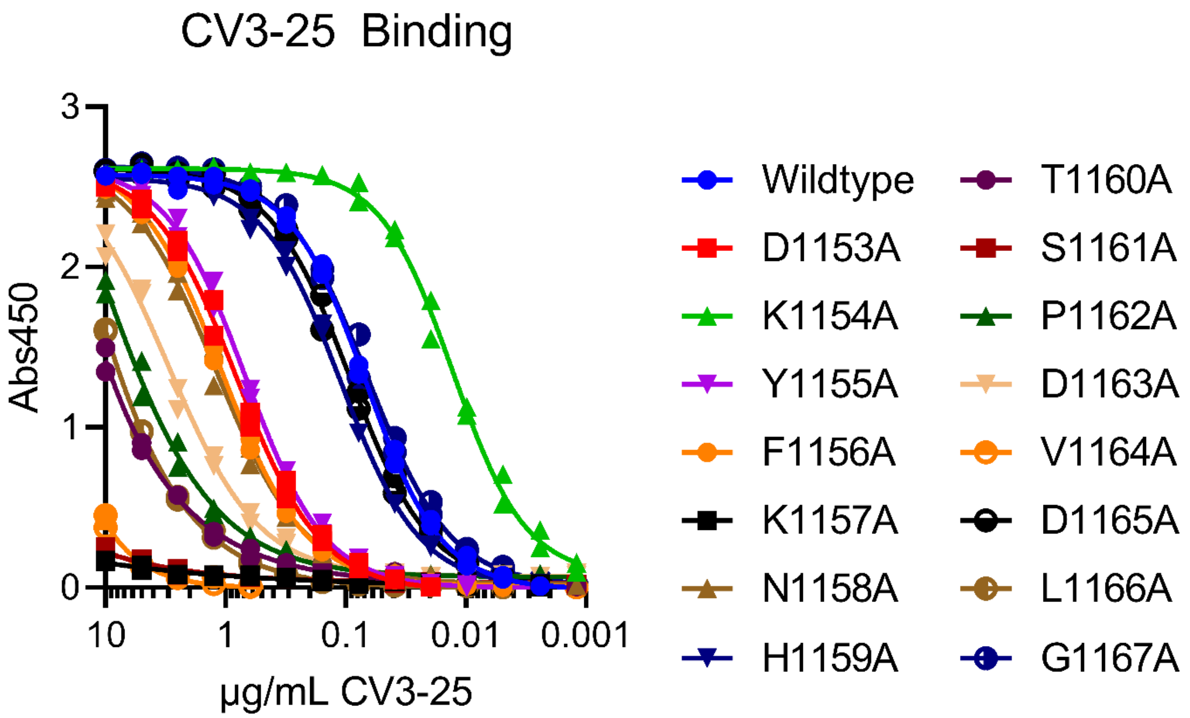

Supplementary Figure 2. CV3-25 binding to linear peptides corresponding to amino acids 1153-1167 of the SARS-CoV-2 spike, where each amino acid was substituted to alanine, was measured by ELISA. One representative example of three independent experiments with two technical replicates is shown.

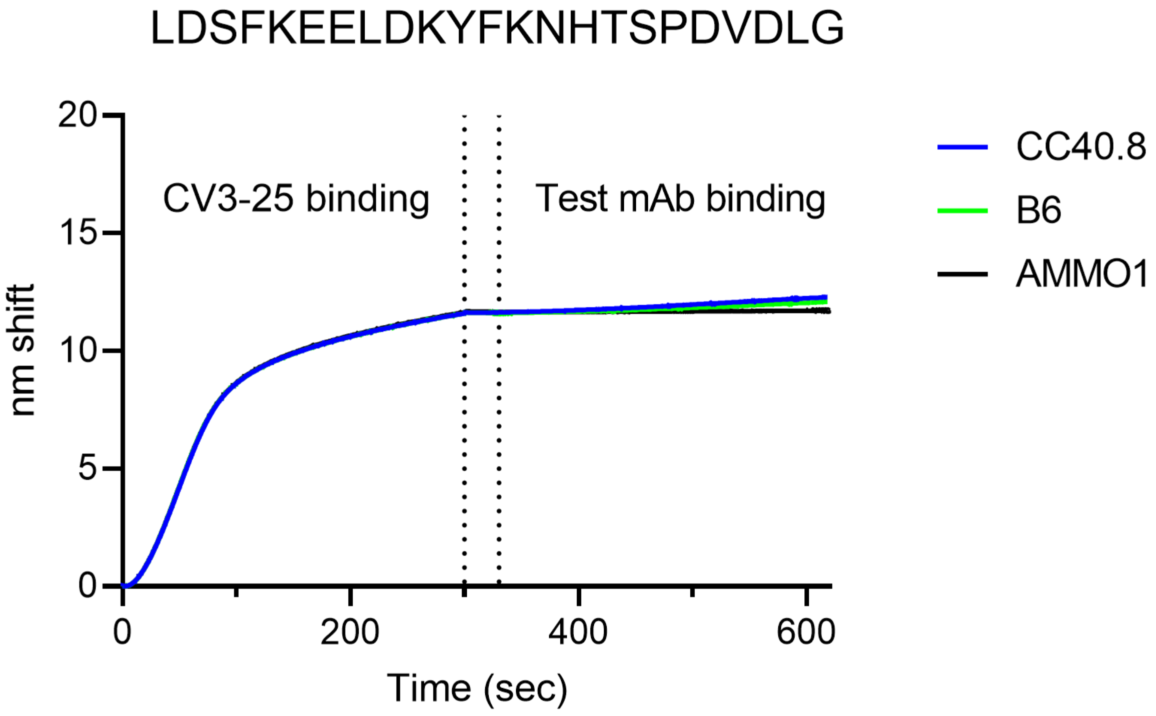

Supplementary Figure 3. CV3-25 competes with stem-helix directed neutralizing mAbs. The indicated SARS-CoV-2 peptide was immobilized on a streptavidin biosensor and immersed into a solution containing CV3-25 for 300s. The sensor was then immersed in kinetics buffer for 30 seconds and then immersed in kinetics buffer containing B6, CC40.8 or AMMO1 as indicated. The dotted lines demarcate the initial binding, baseline, and second binding steps.

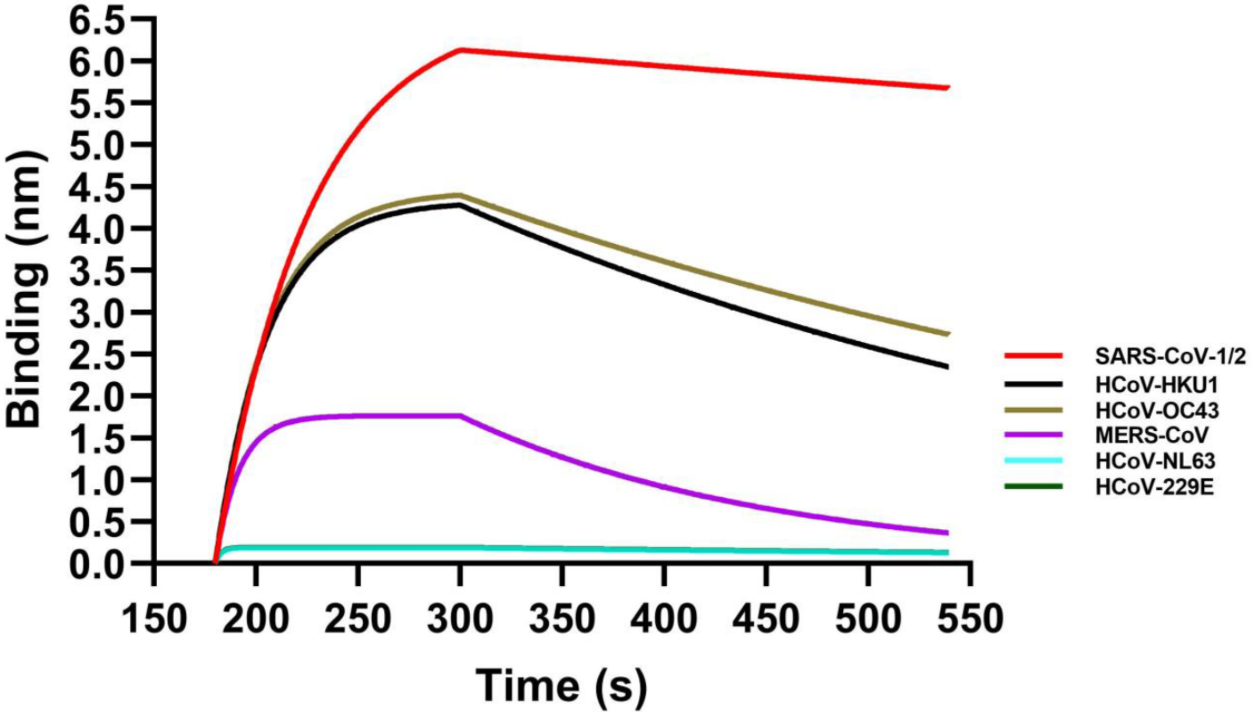

Supplementary Figure 4. CV3-25 binds to linear peptides from diverse Beta CoVs as measured by BLI. Binding of CV3-25 to linear stem helix-derived peptides from the indicated CoVs was measured by BLI.

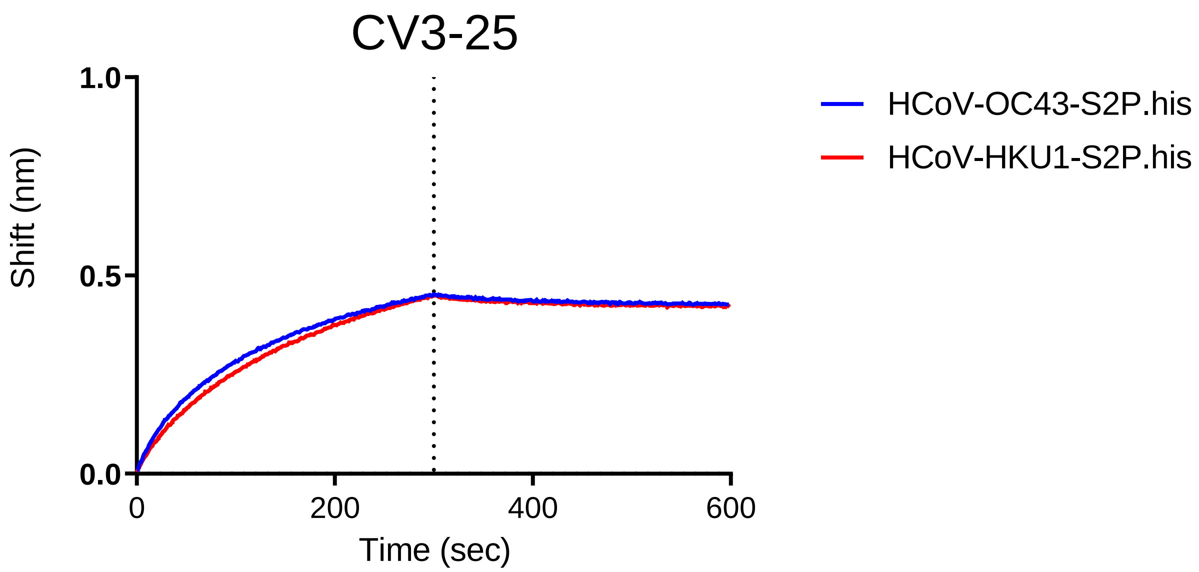

Supplementary Figure 5. CV3-25 binds to stabilized spike ectodomains from HCoV-OC43 and HCoV-HKU1 as measured by BLI as indicated.

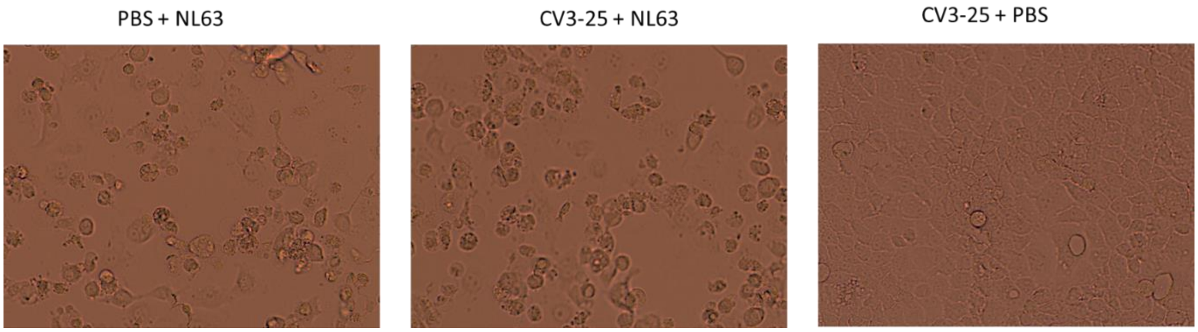

Supplementary Figure 6. CV3-25 does not neutralize HCoV-NL63. LLC-MK2 cells were incubated with $50XTCID_{50}$ NL63 with PBS or 50X $TCID_{50}$ NL63 plus 400 µg/ml CV3-25, or with 400 µg/ml CV3-25 in PBS as indicated. 8 days later the cells were examined for cytopathic effects on a light microscope. Representative images from one of four wells for each condition are shown.

1. Severe acute respiratory syndrome coronavirus 2 isolate 2019-nCoV WHU01 MN988668	L D K Y F K N H T S P D V D L
2. SARS coronavirus Tor2 NC_004718.3	. . . .
3. Bat SARS-like coronavirus WIV1 KF367457.1	. . . .
4. Bat SARS coronavirus Rm1 DQ412043.1	. . . .
5. Bat SARS coronavirus Rp3 DQ071615.1	. . . .
6. Bat SARS coronavirus HKU3-1 DQ022305.2	. . . .
7. Bat SARS coronavirus Rf1 DQ412042.1	. . . .
8. Bat coronavirus strain 16BO133 KY938558	. . . .
9. Bat SARS-like coronavirus isolate Rf4092 KY417145.1	. . . .
10. Bat SARS-like coronavirus RsSHC014 KC881005.1	. . . .
11. SARS-like coronavirus WIV16 KT444582.1	. . . .
12. Bat coronavirus RaTG13 GenBank: MN996532.2	. . . .
13. Pangolin coronavirus isolate PCoV_GX-P4L MT040333	. . . .
14. Pangolin coronavirus isolate PCoV_GX-P5L MT040335.1	. . . .
15. Bat SARS-like coronavirus isolate bat-SL-CoVZXC21 MG772934	. . . . I . .
16. Bat SARS-like coronavirus isolate bat-SL-CoVZC45 MG772933.1	. . . . I . .
17. Severe acute respiratory syndrome-related coronavirus Rc-o319 LC556375.1	. . . . I . .

Supplementary Figure 7. Alignment of the linear epitope recognized by CV3-25 from several bat and pangolin Sarbecoviruses. The name of the isolate and the Genbank accession number is provided for each CoV. Dots represent identity with the Wuhan-Hu-1 isolate of SARS-CoV-2 (boxed in yellow). Single letter amino acid codes represent mismatches.

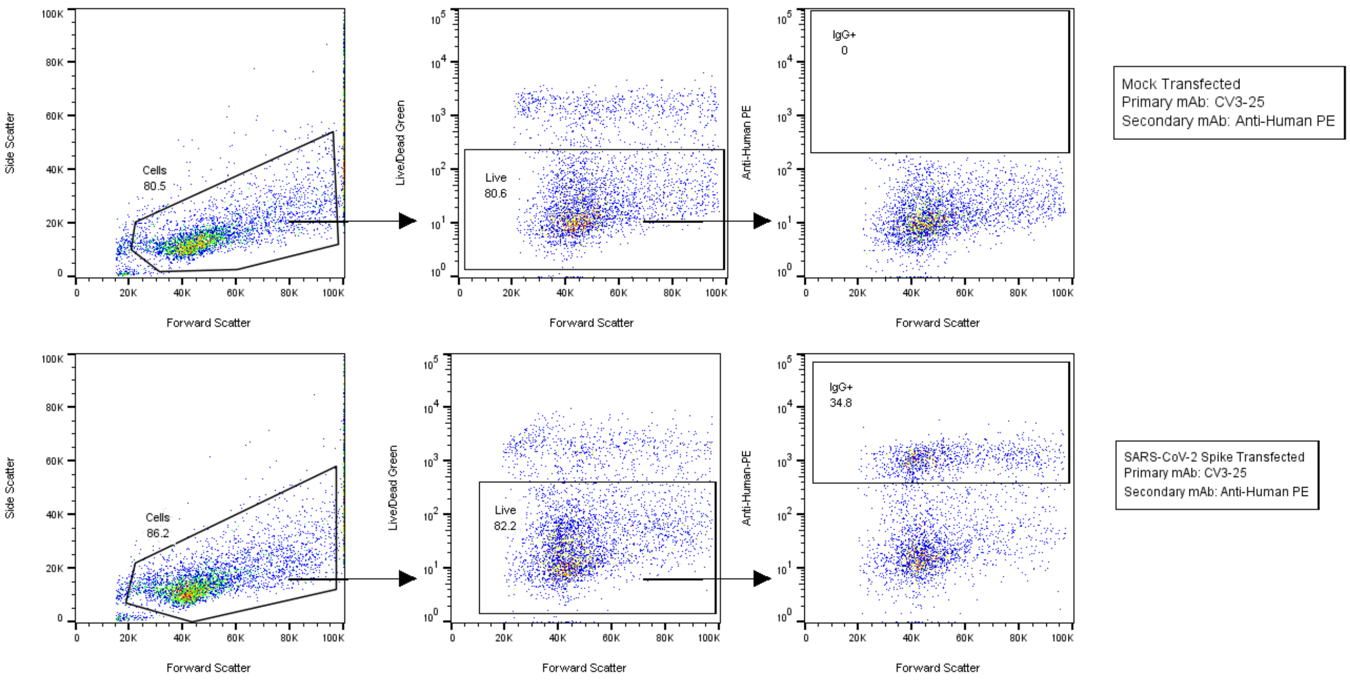

Supplementary Figure 8. Example gating strategy to measure mAb binding to surface expressed CoV spike proteins. See methods for transfection and staining details. Summary data is shown in Figures 5 e-j.

Supplementary Table 1. IC_{50} values of mAb neutralization

mAb	Wuhan-Hu1	Alpha	Beta	Gamma	Delta	Omicron	WIV1
CV3-25	0.32	0.23	0.12	0.47	0.25	0.18	0.71
CV30	.02	.02	0.28	0.12	.01	>5	>50

Values shaded in red show a >5 fold reduction in potency relative to Wuhan-Hu1

Supplementary Table 2. CV3-25 binding kinetics

	K_D (M)X10^{-9}	k_{on} (1/Ms)X10^{5}	K_{on} error (1/Ms)X10^{3}	K_{off} (1/s)X10^{-4}	K_{off} error (1/s)X10^{-5}
SARS-CoV-2 stem helix peptide	5.23	0.30	5.80	1.59	1.24
SARS-CoV-2 S6P	0.66	5.91	4.23	3.89	0.72
HKU1 stem helix peptide	30.98	0.23	0.83	6.76	0.91
OC43 stem helix peptide	39.33	0.17	0.69	6.13	1.00
MERS-CoV-2 stem helix peptide	20.40	0.33	0.69	6.69	0.92
