# Supplementary material for: Structural definition of a pan-sarbecovirus neutralizing epitope on the spike S2 subunit
Source: Commun Biol. 2022 Apr 11;5:342. doi: 10.1038/s42003-022-03262-7 (PMC9001700; doi:10.1038/s42003-022-03262-7)
Supplement: Supplementary file 3 — Description of Additional Supplementary Files [file 42003_2022_3262_MOESM3_ESM.pdf]

Description of Additional Supplementary Files

File name: Supplementary Data 1
Description: Source data for Figure 1: Pseudovirus assay (unprocessed and normalized data).

File name: Supplementary Data 2
Description: Source Data for Figure 2: ELISA data for panels g and h (unprocessed and normalized data.

File name: Supplementary Data 3
Description: Source Data for Figure 5: ELISA data for panels a-d, cell surface-expressed spike staining for panels e-j, OC43 neutralization data for panels k and l, and MERS-CoV neutralization data for panel m (unprocessed and normalized data).
