# Supplementary material for: Structural definition of a pan-sarbecovirus neutralizing epitope on the spike S2 subunit
Source: Commun Biol. 2022 Apr 11;5:342. doi: 10.1038/s42003-022-03262-7 (PMC9001700; doi:10.1038/s42003-022-03262-7)
Supplement: Supplementary file 7 — Reporting Summary [file 42003_2022_3262_MOESM7_ESM.pdf]

nature portfolio

Corresponding author(s): Andrew McGuire, Marie Pancera, Leonidas Stamatatos

Last updated by author(s): Mar 4, 2022

Reporting Summary

Nature Portfolio wishes to improve the reproducibility of the work that we publish. This form provides structure for consistency and transparency in reporting. For further information on Nature Portfolio policies, see our Editorial Policies and the Editorial Policy Checklist.

Statistics

For all statistical analyses, confirm that the following items are present in the figure legend, table legend, main text, or Methods section.

n/a	Confirmed	
☐	☒	The exact sample size (n) for each experimental group/condition, given as a discrete number and unit of measurement
☐	☒	A statement on whether measurements were taken from distinct samples or whether the same sample was measured repeatedly
☒	☐	The statistical test(s) used AND whether they are one- or two-sided<br>*Only common tests should be described solely by name; describe more complex techniques in the Methods section.*
☒	☐	A description of all covariates tested
☒	☐	A description of any assumptions or corrections, such as tests of normality and adjustment for multiple comparisons
☒	☐	A full description of the statistical parameters including central tendency (e.g. means) or other basic estimates (e.g. regression coefficient) AND variation (e.g. standard deviation) or associated estimates of uncertainty (e.g. confidence intervals)
☒	☐	For null hypothesis testing, the test statistic (e.g. F, t, r) with confidence intervals, effect sizes, degrees of freedom and P value noted<br>*Give P values as exact values whenever suitable.*
☒	☐	For Bayesian analysis, information on the choice of priors and Markov chain Monte Carlo settings
☒	☐	For hierarchical and complex designs, identification of the appropriate level for tests and full reporting of outcomes
☒	☐	Estimates of effect sizes (e.g. Cohen's d, Pearson's r), indicating how they were calculated

Our web collection on statistics for biologists contains articles on many of the points above.

Software and code

Policy information about availability of computer code

Data collection	nsEM Particles were picked using DoGPicker via Appion and processed in RELION 3.0
Data analysis	X-ray crystallography: the following description of the software used is found in the methods section: "The data set was processed using XDS and data reduction was performed using AIMLESS in CCP4 to a resolution of 1.74Å. Initial phases were solved by molecular replacement using Phaser in Phenix with a search model of Fab 4AB007 (PDBid: 5MVZ) divided into Fv and Fc portions. Model building was completed using COOT and refinement was performed in Phenix with the final refinement run through the PDB_REDO server". The appropriate references are provided in the methods section.<br>nsEM: Segmentation for illustrations was performed using Segger in USCF Chimera. The appropriate references are provided in the methods section.

For manuscripts utilizing custom algorithms or software that are central to the research but not yet described in published literature, software must be made available to editors and reviewers. We strongly encourage code deposition in a community repository (e.g. GitHub). See the Nature Portfolio guidelines for submitting code & software for further information.

Data

Policy information about availability of data

All manuscripts must include a data availability statement. This statement should provide the following information, where applicable:

- Accession codes, unique identifiers, or web links for publicly available datasets
- A description of any restrictions on data availability
- For clinical datasets or third party data, please ensure that the statement adheres to our policy

All data are available in the manuscript or the supplementary material. TThe CV3-25/peptide structure has been deposited in the PDB (7RAQ). The negative stain EM

map of CV3-25 IgG in complex with SARS-CoV-2 6P-D614G S protein has been deposited to the Electron Microscopy Data Bank under accession code EMD-25498. All reagents generated in this study are available upon request through Material Transfer Agreements. pTT3-derived plasmids and 293-6E cells require a license from the National Research Council (Canada)

Field-specific reporting

Please select the one below that is the best fit for your research. If you are not sure, read the appropriate sections before making your selection.

☒ Life sciences ☐ Behavioural & social sciences ☐ Ecological, evolutionary & environmental sciences

For a reference copy of the document with all sections, see nature.com/documents/nr-reporting-summary-flat.pdf

Life sciences study design

All studies must disclose on these points even when the disclosure is negative.

Sample size	N/A
Data exclusions	No data were excluded from the analysis.
Replication	All binding and neutralization assays were repeated at least twice unless otherwise noted.
Randomization	N/A
Blinding	N/A

Reporting for specific materials, systems and methods

We require information from authors about some types of materials, experimental systems and methods used in many studies. Here, indicate whether each material, system or method listed is relevant to your study. If you are not sure if a list item applies to your research, read the appropriate section before selecting a response.

Materials & experimental systems

n/a	Involved in the study	
☐	☒	Antibodies
☐	☒	Eukaryotic cell lines
☒	☐	Palaeontology and archaeology
☒	☐	Animals and other organisms
☒	☐	Human research participants
☒	☐	Clinical data
☒	☐	Dual use research of concern

Methods

n/a	Involved in the study	
☒	☐	ChIP-seq
☐	☒	Flow cytometry
☒	☐	MRI-based neuroimaging

Antibodies

Antibodies used	Goat anti-Human Ig-HRP (Southern Biotech, Cat# 2010-05), AffiniPure Fab fragment goat anti-human IgG (Jackson Immunoresearch Cat# 109-117-008)
Validation	N/A

Eukaryotic cell lines

Policy information about cell lines

Cell line source(s)	293-6E (National Research Council of Canada), 293T (ATCC), HEK-293T-hACE2 (BEI resources), HCT-8 (ATCC), LCC-MK2 (ATCC), Huh7 (Dr. Ram Savan, University of Washington).
Authentication	None of the cell lines used were authenticated.
Mycoplasma contamination	None of the cell lines used were tested for mycoplasma contamination.
Commonly misidentified lines (See ICLAC register)	N/A

Flow Cytometry

Plots

Confirm that:

☒ The axis labels state the marker and fluorochrome used (e.g. CD4-FITC).

☒ The axis scales are clearly visible. Include numbers along axes only for bottom left plot of group (a 'group' is an analysis of identical markers).

☒ All plots are contour plots with outliers or pseudocolor plots.

☒ A numerical value for number of cells or percentage (with statistics) is provided.

Methodology

Sample preparation	Spike-transfected or mock-transfected 293T cells were resuspended at 4x106 cells/ml in FACS buffer and 50µl was added to each well of the 96 well plate. mAb-cell mixture was incubated for 30 minutes on ice. The plates were then washed once with 200µl of FACS buffer and stained with of PE-conjugated , AffiniPure Fab fragment goat anti-human IgG (Jackson Immunoresearch Cat# 109-117-008) at a 1:100 dilution and live/dead green fluorescent reactive dye (Thermo Fisher Cat# L34970) at a 1:1000 dilution in 50 µl/well of 1X PBS. The staining reaction was incubated for 20 minutes in the dark on ice. The plates were then washed once with 200µl of FACS buffer and fixed with 50 µl of 10% formalin. The plates were centrifuged, and the formalin was removed and replaced with 250µl of FACS buffer
Instrument	Guava easyCyte 5HT Flow Cytometer (Luminex)
Software	GuavaSoft Software (collection), FlowJo (Analysis)
Cell population abundance	N/A
Gating strategy	PE positivity was determined by first determining the PE flouresence intensity of the negative population by staining mock transfected with a primary (anti-SARS-CoV-2 spike human mAb) and a secondary anti-human PE mAb). Transected cells expressing the SARS-CoV spike were scored as PE positive if the PE fluoresence was above the negative population.

☒ Tick this box to confirm that a figure exemplifying the gating strategy is provided in the Supplementary Information.
